# Inhibiting the Progression of Human Retinoblastoma Cell by Downregulation of MMP-2/MMP-9 Using Short Hairpin RNAs (shRNAs) In Vitro

**DOI:** 10.1155/2020/4912347

**Published:** 2020-05-13

**Authors:** Nana Meng, Zhi Zhao, Chunhe Shi, Leizhou Xia

**Affiliations:** ^1^Department of Ophthalmology, Affiliated People's Hospital, Jiangsu University, Zhenjiang 212002, China; ^2^Zhenjiang Kangfu Eye Hospital, Zhenjiang 212002, China; ^3^Department of General Surgery, Affiliated People's Hospital, Jiangsu University, Zhenjiang 212002, China

## Abstract

**Objective:**

To investigate the effect of downregulated matrix metalloproteinases (MMPs) gene on the proliferation, apoptosis, cell cycle, migration, and invasion of human retinoblastoma (RB) cell line in vitro.

**Methods:**

Small hairpin RNA (shRNA) targeting MMP-2/MMP-9 was designed and transfected into WER1-Rb-1 cells. 48 hours after transfection, qRT-PCR and western blot technique were used to investigate the inhibitory effect of MMP-2 and MMP-9 shRNAs. Cell viability was examined by 3-(4,5-dimethylthiazol-2-yl)-2,5-diphenyltetrazolium bromide (MTT) assay. Cell cycle arrest was detected using a flow cytometer while apoptosis was tested with Annexin V/PI kit. Transwell chamber assay was performed to detect the migration and invasion ability of the WER1-Rb-1 cells.

**Results:**

After transfection of MMP-2/MMP-9 shRNA, there was a significant decrease in the expressions of both mRNA and protein in the shRNA groups compared with the negative and vector controls. The results of MTT assay suggested that the cell viability was significantly decreased in shRNA groups (*p<*0.05). Cell apoptosis also increased significantly in shRNA groups compared with the negative and vector controls (*p<*0.05). The flow cytometer analysis proved that the proportion of the G1 phase increased and the proportion of the G0 phase reduced significantly by the transfection of MMP-2/MMP-9 shRNA (*p<*0.05). The migration and invasion ability were also significantly decreased in the groups of MMP-2/MMP-9 shRNA (*p<*0.05).

**Conclusions:**

Cell viability, migration, and invasion ability of RB cells are inhibited, and apoptosis is induced after downregulation of MMP-2/MMP-9 through RNA interference. MMP-2 and MMP-9 may be potential targets in the gene therapy of RB.

## 1. Introduction

Developing from the immature cells of retina, retinoblastoma (RB), as the most common primary intraocular malignancy of childhood, is a rare form of ocular malignant tumor [1–3] with an incidence of approximately 1/15,000–20,000 live births worldwide [4]. If diagnosed early, RB can be effectively treated by a reasonable therapy such as intravenous chemotherapy together with focal therapy (laser cryotherapy). However, most advanced cases will be involved in a distant invasion or metastasis due to the limited available diagnostic approaches, which may result in an elevated mortality rate [5]. Since the treatment of RB is mainly aimed at survival and the globe salvage and visional preservation are secondary goals, there is a critical need for the development of new targeted therapies to decrease the invasion and metastasis of RB.

Matrix metalloproteinases (MMPs) can degrade the base membrane, which is a crucial step in the tumor invasion and subsequent distant metastasis [6]. MMPs belong to a family of zinc ion-dependent endopeptidases among which MMP-2 and MMP-9 are considered to be indispensable for the degradation of the main components of extracellular matrix (ECM) [7]. Recently, the high expression of MMP-2 and MMP-9 has been confirmed in various malignant tumors, including pancreatic cancer [8], oral squamous cell carcinoma [9], cervical carcinoma [10], and RB as well [11–13]. The tissue inhibitors of metalloproteinases (TIMPs) are responsible for regulating the enzymatic activities of MMPs [14]. Accordingly, downregulation of the expression of the MMP-2/MMP-9 gene may be beneficial to inhibit the development and invasion of RB. However, studies on how to inhibit RB cells by silencing the MMP-2/MMP-9 gene are limited. In the present study, the expression of the MMP-2/MMP-9 gene was downregulated by small hairpin RNA (shRNA) to study its effect on RB cell proliferation, apoptosis, cell cycle, migration, and invasion, and furthermore to explore the potential clinical significance of blocking MMP-2 and MMP-9 gene expression as an antitumor therapeutic target.

## 2. Materials and Methods

### 2.1. Retinoblastoma Cells

A human RB cell line, WER1-Rb-1, was purchased from the cell bank of Shanghai Institutes for Biological Sciences (Shanghai, China). All cells were cultured in RPMI-1640 medium (Gibco, Grand Island, NY, USA) with 10% fetal bovine serum (FBS, Sigma, St. Louis, Missouri, USA), 100 U/ml penicillin, and 100 mg/ml streptomycin, and maintained at 37°C/5% CO_2_. The medium was changed every 3 days, and cells were subcultured every 2-3 days and observed daily.

### 2.2. Silence of MMP-2/MMP-9 Expression in WER1-Rb-1 Cells

To suppress the MMP-2/MMP-9 expression in RB cells, a powerful gene-specific silencing technique utilizing the RNA interference (RNAi) mechanism was used. The sequence-verified plasmid vector (plenty GFP Puro) based on the short hairpin RNA library targeting human MMP-2/MMP-9 (MMP-2/MMP-9-shRNA) was purchased from GenePharma Co. (Shanghai, China). Short hairpin RNAs (shRNAs) were processed into siRNAs intracellularly, leading to a specific loss of function of MMP-2/MMP-9 gene effectively. In the experiments of transfection, WER1-Rb-1 cells were seeded into a 24-well plate and cultured in RPMI-1640 without antibiotics. And then, the transfection was performed using Lipofectamine 2000 (Invitrogen, Carlsbad, CA, USA) according to the manufacturer's instructions.

### 2.3. RNA Extraction and Quantitative Real-Time PCR (qRT-PCR)

Total RNA was isolated from WER1-Rb-1 cell with the TRIzol reagent (Ambion) according to the manufacturer's protocol. Using the M-MLV1 reverse transcription kit (Takara, Dalian, China), total RNA was reversely transcribed to cDNA, and then quantified with Universal SYBR Green Ι detection assays (Bioteke Corporation) using an Applied Biosystems 7900 HT system (Funglyn Biotech Incorporated). The primers used in this study were as follows: MMP-2, 5-TGATGGCATCGCTCAGATCC-3 and 5-GGCCTCGTATACCGCATCAA-3; MMP-9, 5-GGACAAGCTCTTCGGCTTCT-3 and 5-TCGCTGGTACAGGTCGAGTA-3; and GAPDH, 5-CTCCTCCTGGCCTCGCTGT-3 and 5-GCTGTCACCTTCACCGTTCC-3.

### 2.4. Western Blotting

WER1-Rb-1 cells were harvested and lysed with RIPA buffer (Beyotime, Jiangsu, China). Protein concentrations were determined by a BCA protein assay kit (Beyotime) according to the manufacturer's instructions. Proteins were separated using 12% SDS-polyacrylamide gels and transferred from the gel to nitrocellulose membranes. Membranes were blocked with 5% nonfat milk and then exposed to the following primary antibodies: MMP-2 (1 : 1000, sc-13594, Santa Cruz), MMP-9 (1 : 1000, sc-6841, Santa Cruz), and anti-GAPDH (1 : 1000, sc-365062, Santa Cruz). The blots were rinsed with 0.1% TBST and incubated with horseradish peroxidase-(HRP-) conjugated corresponding secondary antibody (1 : 5000) for 1 h at room temperature. GAPDH was used as a control, and all the antibodies were purchased from Santa Cruz Biotechnology.

### 2.5. Cell Viability

3-(4,5-Dimethylthiazol-2-yl)-2,5-diphenyltetrazolium bromide (MTT) assay was applied to determine cell viability. In brief, 3 *∗* 10^3^ transfected WER1-Rb-1 cells were plated into 96-well plates and cultured for 24–72 h. At the indicated time, 20 *μ*l MTT solution (3 mg/ml, Beyotime) was added to each well, followed by an additional culture for 4 h. Then, the medium was removed, and 150 *μ*l dimethyl sulfoxide (DMSO, Sigma-Aldrich) was added to each well for dissolving MTT formazan crystals. Optical density (OD) was detected at the wavelength of 490 nm using a microplate spectrometer (Thermo Scientific, Waltham, USA).

### 2.6. Cell Cycle Distribution and Analysis of Apoptosis

WER1-Rb-1 cells were harvested and washed with PBS 48 h after transfection. Then, 5 *∗* 10^4^ cells were resuspended in 200 *μ*l of binding buffer containing 5 *μ*l of Annexin V-fluorescein isothiocyanate (FITC) and 5 *μ*l of propidium iodide (PI). The cell cycle test was performed using an FACS-Calibur™ flow cytometer (BD Biosciences, San Jose, CA, USA) and was analyzed by CellQuest software (BD Biosciences). Cell apoptosis was determined with an Annexin V-FITC Apoptosis Detection kit (Beyotime). The data were analyzed with FlowJo v5.7.2 software (BD Biosciences).

### 2.7. Migration and Invasion Assay

Transwell chamber was applied to perform the migration and invasion assay. Matrigel was used to precoat the membrane of transwell to simulate a matrix barrier for the invasion assay. The transfected cells growing in the log phase were seeded on the upper champers at a density of 1 *∗* 10^5^ cells/well. To stimulate cell invasion, 600 *μ*l medium with 20% FBS was added to the lower chamber. After 24 h of incubation, cells which migrated to the lower chamber were fixed with paraformaldehyde for 30 min and stained with 0.1% crystal violet for 15–30 min. The images of cells were photographed with an inverted microscope (Olympus) at ×100 magnification, and the cell numbers were counted in five random fields per membrane.

### 2.8. Statistical Analysis

GraphPad Prism 6 (GraphPad Software, Inc, San Diego, CA, USA) software was applied for statistical analysis. Comparisons between the groups were analyzed with two-tailed Student's *t*-test or one-way ANOVA. A *p* value <0.05 was considered statistically significant in all analyses. All data are represented as the mean ± SEM (standard error of the mean) from at least three independent experiments, as indicated with the significance score (^*∗*^*<*0.05; ^*∗∗*^*<*0.01; ^*∗∗∗*^*<*0.001; ^*∗∗∗∗*^*<*0.0001) in the figure legends.

## 3. Results

### 3.1. MMP-2/MMP-9 Downregulated by RNA Interference in WER1-Rb-1 Cells

**Figure 1:**
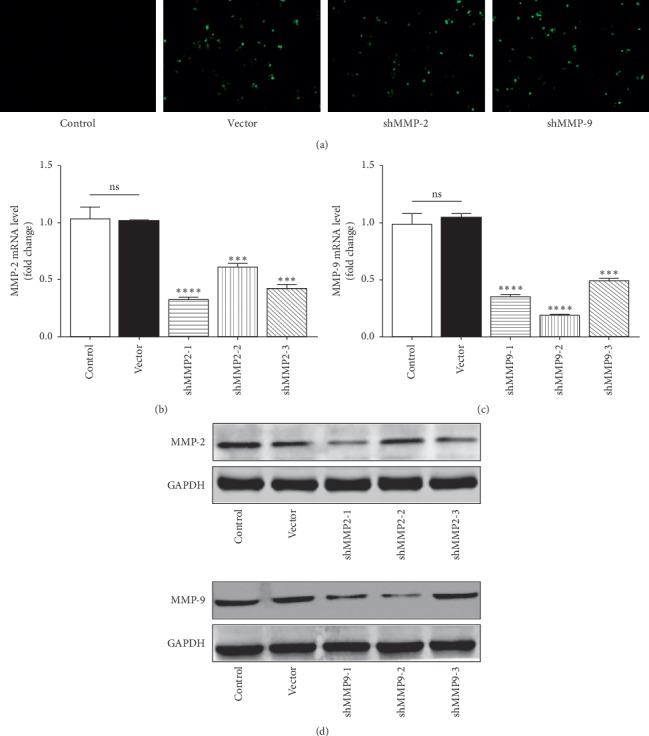
Confirmation of MMP-2/MMP-9 knockdown by RNAi in WER1-Rb-1 cells. (a) Representative images of WER1-Rb-1 cells after transfection under fluorescence microscopy. (b) Decreased MMP-2 mRNA level after MMP-2 shRNA transfection. (c) Decreased MMP-9 mRNA level after MMP-9 shRNA transfection. (d) Representative WB images of MMP-2/MMP-9 for each group with GAPDH serving as a loading control. ^*∗∗∗*^*p<*0.001, ^*∗∗∗∗*^*p<*0.0001.

The shRNA sequences for MMP-2/MMP-9 were fused with a green fluorescent protein (GFP) cDNA by using the plasmids in this study. Therefore, the transfected WER1-Rb-1 cells exhibited strong green fluorescence under a fluorescence microscope, while there was no fluorescence for the control group (Figure 1(a)). Additionally, the time point of the most significant transfection efficacy was 48 hours after transfection in this study. To get the optimal RNA interference effect, three different sense sequences targeting MMP-2/MMP-9 were constructed, respectively (MMP-2: CCCTTCTTGTTCAATGGCA, ACACTAAAGAAGATGCAGA, AGGTGATCTTGACC-AGAAT; MMP-9: CCGAGCTGACTCGACGGTG, TGGTGCGCTACCACCTCGA, ACGC-ACGACGTCTTCCAGT). To investigate MMP-2/MMP-9 expression in WER1-Rb-1 cells after transfection with different shRNAs, qRT-PCR was performed. As shown in Figures 1(b) and 1(c), shRNA-1 for MMP-2 (shMMP2-1) and shRNA-2 for MMP-9 (shMMP9-2) were the most effective, respectively. Moreover, we got consistent results for the expression of MMP-2/MMP-9 protein after transfection from WB (Figure 1(d)) results. Simultaneously, the mRNA and the protein level of MMP-2/MMP-9 were almost identical between the control group and vector group (Figures 1(b)–1(d)). Accordingly, shMMP2-1 and shMMP9-2 were chosen for the further experiments.

### 3.2. Downregulation of MMP-2/MMP-9 Inhibits WER1-Rb-1 Cell Viability

**Figure 2:**
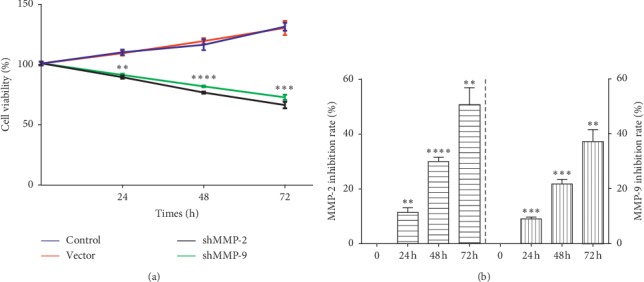
MMP-2/MMP-9 knockdown inhibits the proliferation of WER1-Rb-1 cells. (a) MTT assay results of each group at different time points after transfection. (b) Inhibition rate of shMMP-2/shMMP-9 significantly increased with time going. ^*∗∗*^*p<*0.01, ^*∗∗∗*^*p<*0.001, ^*∗∗∗∗*^*p<*0.0001.

The MTT assay showed that there was no difference for cell viability at any indicated time point between the control group and vector group, suggesting that the blank vector had no effect on cell proliferation. However, downregulation of MMP-2/MMP-9 through shRNA transfection remarkably decreased the WER1-Rb-1 cell viability (24 h, vector versus shMMP-2/shMMP-9, *p=*0.0022*/*0.002; 48 h, vector versus shMMP-2/shMMP-9, *p<*0.0001 for both; 72 h, vector versus shMMP-2/shMMP-9, *p=*0.0003*/*0.0001; Figure 2(a)). Furthermore, the inhibition rate of MMP-2/MMP-9 appeared to increase in a time-dependent manner following transfection (Figure 2(b)).

### 3.3. Inhibition of MMP-2/MMP-9 Affected the Cell Cycle Arrest and Increased Apoptosis of WER1-Rb-1 Cell

**Figure 3:**
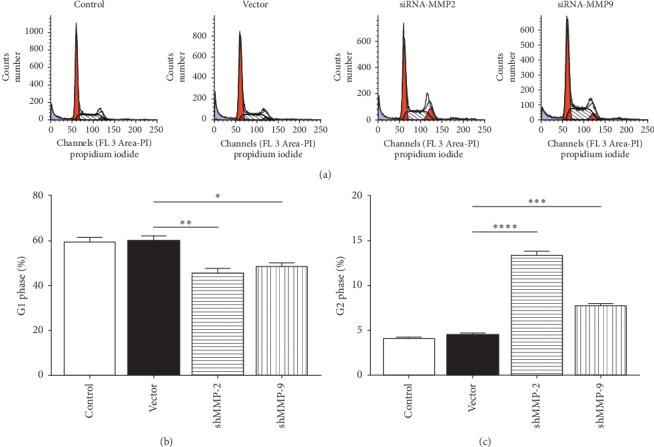
Silence of MMP-2/MMP-9-altered cell cycle distribution of WER1-Rb-1 cells. (a) Representative FACS images of cell cycle for each group. (b, c) Normalized proportion of G1/G2 phase cells, plotted as mean ± SEM of triplicates per group. ^*∗*^*p<*0.05, ^*∗∗*^*p<*0.01, ^*∗∗∗*^*p<*0.001, ^*∗∗∗∗*^*p<*0.0001.

**Figure 4:**
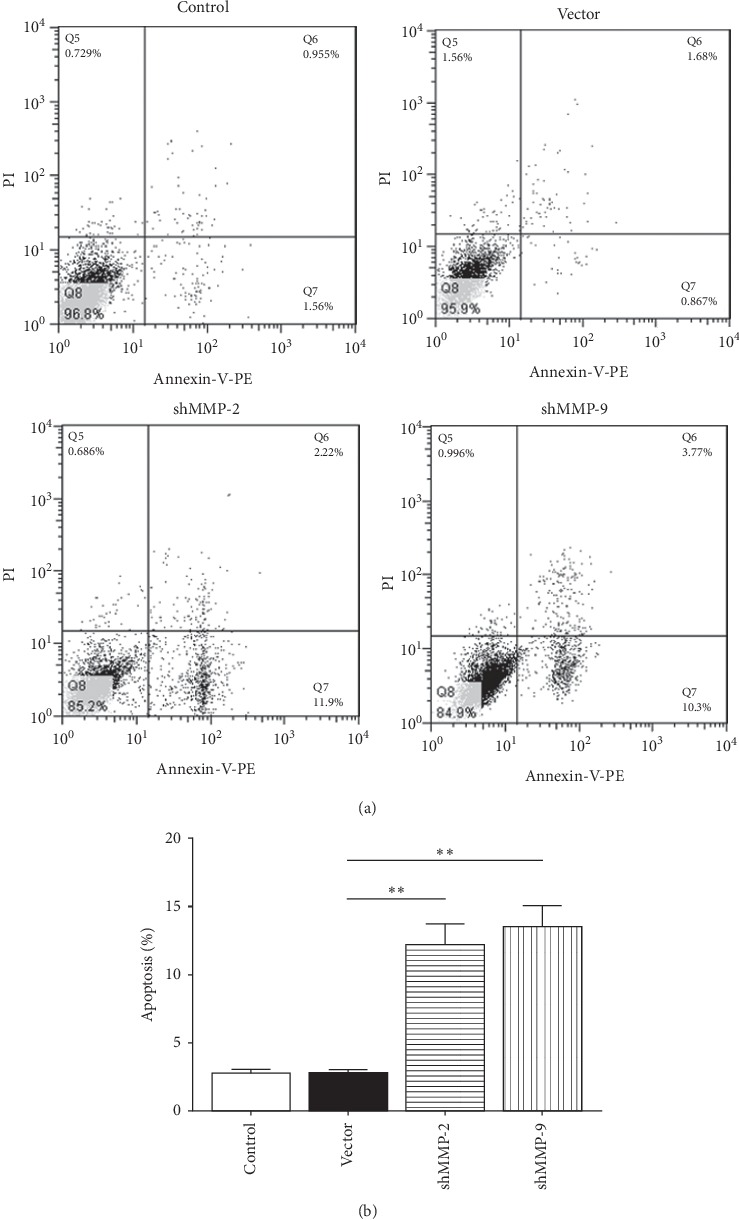
MMP-2/MMP-9 inhibition induces the apoptosis of WER1-Rb-1 cells. (a) Representative FACS images based on Annexin-V-PE and PI staining for each group. (b) Apoptosis was determined in WER1-Rb-1 cells transfected with MMP-2 and MMP-9 shRNA. ^*∗∗*^*p<*0.01.

FACS was conducted to determine the effect of downregulated MMP-2/MMP-9 on the cell cycle of WER1-Rb-1 cells. As illustrated in Figures 3(a) and 3(b), the vector transfection did not influence the cell cycle, while transfection of shMMP-2/shMMP-9 after 48 hours significantly decreased the proportion of G1 phase cells compared with the vector group (vector versus shMMP-2, *p=*0.0074; vector versus shMMP-9, *p=*0.0105). Simultaneously, the proportion of G2 phase cells was remarkably increased 48 hours after transfection (vector versus shMMP-2, *p<*0.0001; vector versus shMMP-9, *p=*0.0006; Figures 3(a) and 3(c)). In addition, an FACS analysis showed that cell apoptosis rate was unaffected in the vector group in comparison to the control group, while knockdown of MMP-2/MMP-9 significantly increased the cell apoptosis rate (vector versus shMMP-2, *p=*0.0034; vector versus shMMP-9, *p=*0.0023; Figures 4(a) and 4(b)).

### 3.4. MMP-2/MMP-9 Knockdown Inhibited the Migration and Invasion of WER1-Rb-1 Cell

**Figure 5:**
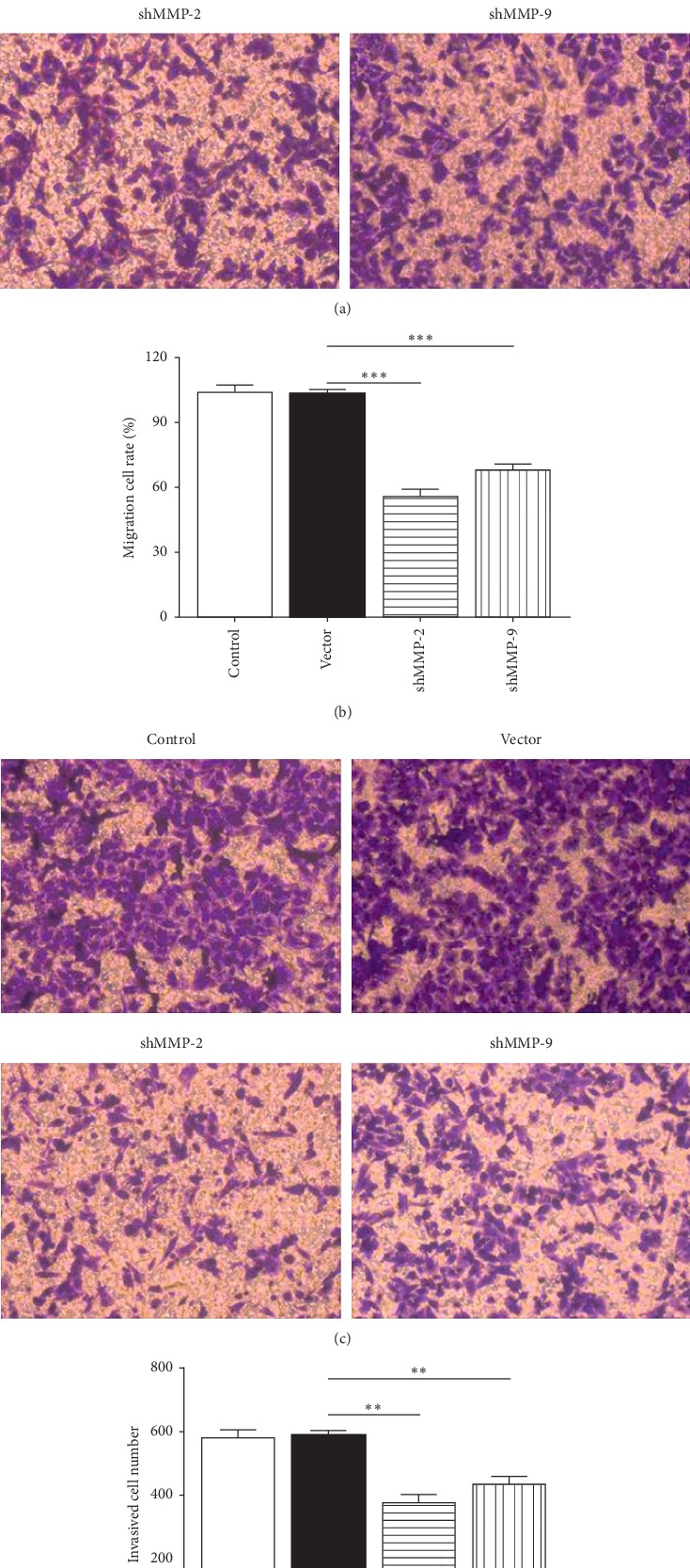
shMMP-2 and shMMP-9 inhibits migration and invasion of WER1-Rb-1 cells. (a, b) MMP-2/MMP-9 knockdown inhibited migration of WER1-Rb-1 cells, as determined by transwell migration assay. (c, d) Transwell migration assay showed that MMP-2/MMP-9 knockdown inhibited invasion of WER1-Rb-1 cells. ^*∗∗*^*p<*0.01, ^*∗∗∗*^*p<*0.001.

To detect the effect of MMP-2/MMP-9 on WER1-Rb-1 cell migration and invasion, transwell assay was performed. Figures 5(a) and 5(b) demonstrated that WER1-Rb-1 cell transfected by MMP-2/MMP-9 shRNA significantly suppressed the cell capacity of migration (vector versus shMMP-2, *p=*0.0003; vector versus shMMP-9, *p=*0.0005). Similarly, we got parallel results for the decreased cell invasion ability after MMP-2/MMP-9 shRNA transfection (vector versus shMMP-2, *p=*0.0021; vector versus shMMP-9, *p=*0.0055; Figures 5(c) and 5(d)). As expected, there was no difference for the cell migration or invasion between the negative control group and vector group.

## 4. Discussion

Enucleation is the critical therapy for intraocular RB [1], which has been proved to be a therapeutic standard for the unilateral RB cases. Currently, more and more novel managements for advanced RB are being tested to minimize the need of enucleation because child survival is still the therapeutic goal with highest priority and prompt removal of affected eyeballs shows the decreasing risk of potential tumor spread [1, 2, 15]. The pathogenesis of tumor spread and metastasis involves a series of complicated steps, among which the degradation of the ECM is supposed to be the crucial one [16, 17]. MMPs are the main proteinases, which are responsible for degrading the majority of protein in base membrane and ECM. Among the components of ECM, gelatinase A and gelatinase B which mostly degrade gelatin and several types of basal membrane collagen have a close positive correlation with MMP-2 and MMP-9, respectively [18]. Except for the degeneration of ECM, MMP-2 and MMP-9 also influence the adherence and motility of tumor cells [19]. Long H's study showed that the expression of MMP-2 and MMP-9 were closely associated with optic nerve invasion and clinical stage of RB, which implied that inhibition of MMP-2 and MMP-9 will be beneficial to the invasion and metastasis of RB [12].

Previous research has proved that MMP-2 and MMP-9 are present in RB cells, and the expression level is significantly higher in RB tissue compared to normal retina [7, 11–13]. In this study, we addressed the effect of downregulated MMP-2/MMP-9 gene on the proliferation, apoptosis, cell cycle, migration, and invasion of WER1-Rb-1 cell line. Besides, we can draw that the decreased cell proliferation of RB attributes to the elevated apoptosis rate from the results of cell cycle and apoptosis analysis. To the best of our knowledge, we are the first to knockdown MMP-2/MMP-9 in RB cell in vitro stably by shRNA gene silencing technique, which was different from the study of Webb et al. in 2017 [13]. For the past decades, RNA interference (RNAi) has been becoming a cogent tool for the silence of gene function in mammalian cells. Among the RNAi technologies, shRNAs can be applied to generate the knockdown cell lines stably, which eliminates the need for repetitive transfection and significantly improves reproducibility of the results compared with the small interfering RNAs (siRNAs) [20]. Consistent with some previous reports [12, 13], our results also indicated that cell viability, migration, and invasion ability of RB cell were inhibited, and apoptosis of RB cell was induced due to downregulation of MMP-2/MMP-9, suggesting that MMP-2 and MMP-9 might be the potential targets for preventing the progression of RB.

**Figure 6:**
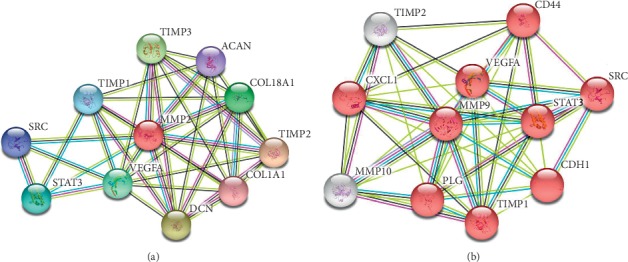
Potential MMP-2 and MMP-9 protein-protein interaction networks adopted from string database, version 11.0. (a) ACAN, aggrecan core protein; COL1A1, collagen alpha-1 (I) chain; COL18A1, collagen alpha-1 (XVIII) chain; DCN, cecorin; SRC, proto-oncogene tyrosine-protein kinase Src; STAT3, signal transducer and activator of transcription 3; TIMP 1/2/3, metalloproteinase inhibitor 1/2/3; VEGFA, vascular endothelial growth factor A. (b) CD44, cluster of differentiation 44; CDH1, cadherin-1; CXCL1, chemokine (C-X-C motif) ligand 1; MMP10, matrix metalloproteinase-10; PLG, plasminogen; SRC, proto-oncogene tyrosine-protein kinase Src; STAT3, signal transducer and activator of transcription 3; TIMP 1/2, metalloproteinase inhibitor 1/2; VEGFA, vascular endothelial growth factor A.

However, the present study still has its limitations. The molecular mechanisms by which MMP-2/MMP-9 which exert their effects on the biological behavior of RB tumor cells were not detected in this study. To get deeper understanding of MMP-2/MMP-9 effects on RB, STRING database (version 11.0) was applied to explore the potential protein-protein interactions (PPI). As demonstrated in the PPI networks of Figures 6(a) and 6(b), many proteins (described in the figure legends in detail) are closely associated with MMP-2/MMP-9. Moreover, possible coexpression, cooccurrence, metabolic pathways, and signal transduction pathways about MMP-2/MMP-2 with other proteins are obtained from the database as well.

In summary, our findings revealed that downregulation of MMP-2/MMP-9 induces the apoptosis and impairs the viability, migration, and invasion of RB tumor cells, providing a potential MMP target therapy in RB patients.

## Data Availability

All data generated or analyzed during this study are included within this article.

## Abbreviations

ECM: Extracellular matrix
FACS: Fluorescence-activated cell sorting
MMP: Matrix metalloproteinase
MTT: 3-(4,5-Dimethylthiazol-2-yl)-2,5-diphenyltetrazolium bromide
PPI: Protein-protein interaction
RB: Retinoblastoma
RNA: Ribonucleic acid
RT-PCR: Reverse transcription polymerase chain reaction
shRNA: small hairpin RNA
siRNA: small interfering RNA
WB: Western blotting.
